# Targeted drug repurposing in medication-related osteonecrosis of the jaw: a review of teriparatide and pentoxifylline/α-tocopherol protocols

**DOI:** 10.3389/fmed.2026.1750238

**Published:** 2026-03-03

**Authors:** Weijia Huang, Jishizhan Chen, Quan Zhou, Azadeh Rezaei

**Affiliations:** 1 Department of Oral and Maxillofacial Surgery, Second Affiliated Hospital, Zhejiang University School of Medicine, Hangzhou, China; 2 Division of Surgery and Interventional Science, Royal Free Hospital, University College London, London, United Kingdom; 3 UCL Mechanical Engineering, Torrington Place, University College London, London, United Kingdom; 4 Department of Pharmacy, Second Affiliated Hospital, Zhejiang University School of Medicine, Hangzhou, China

**Keywords:** anti-cancer drug, drug repurposing, medication-related osteonecrosis of the jaw, pentoxifylline, teriparatide, α-tocopherol

## Abstract

Medication-related osteonecrosis of the jaw (MRONJ) is a severe adverse effect associated with antiresorptive and antiangiogenic treatments commonly prescribed for patients with cancer or osteoporosis. The increasing prescription of these drugs, coupled with the introduction of new anti-cancer medications, has raised concerns regarding the increasing risk of MRONJ. While the precise mechanisms underlying MRONJ remain unclear and effective therapies are still lacking, two repurposed pharmacological protocols—teriparatide (TPTD) and the combination of pentoxifylline (PTX) and α-tocopherol (TOC) [PENTO protocol]—have demonstrated potential therapeutic benefits. However, large-scale clinical evidence remains insufficient. This review evaluates the therapeutic potential of these targeted drug repurposing protocols, exploring their mechanisms of action in MRONJ management and proposing a clinical application protocol for both prevention and treatment. This study also highlights the potential of drug repurposing as a rapid and cost-effective approach for MRONJ management, particularly for patients with cancer, and emphasises the need for further research on personalised and localised management strategies. Nevertheless, the current evidence base is limited by small sample sizes, heterogeneous patient populations, non-randomised study designs, and inconsistent outcome measures, precluding definitive conclusions regarding efficacy and optimal clinical use.

## Graphical abstract

**Figure:**
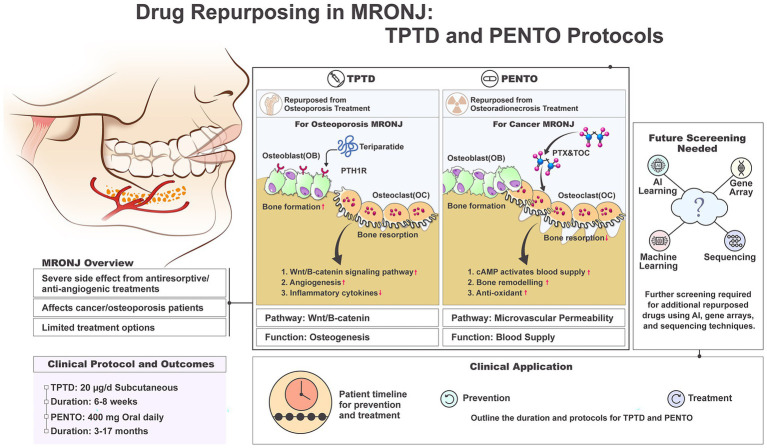

## 1 Introduction

Since the first reported case in 2003, the management of medication-related osteonecrosis of the jaw (MRONJ) remains an ongoing clinical challenge (1). MRONJ is primarily associated with antiresorptive and antiangiogenic medications, commonly prescribed to patients with cancer to prevent skeletal-related complications and to patients with osteoporosis to reduce bone resorption (2). A multicentre study revealed that antiresorptive agents, including bisphosphonates (BPs) and denosumab, are most frequently implicated in MRONJ (3). Despite ongoing research, the mechanisms underlying MRONJ remain elusive, and effective therapies are lacking, making it a significant clinical hurdle (4).

With the increasing use of these medications, a significant number of individuals are at risk for MRONJ. The estimated prevalence ranges from 5 to 10% in patients with cancer and from 0.05 to 5% in patients with osteoporosis (5, 6). The ratio of MRONJ incidence between patients with cancer and those with osteoporosis is reported to range from 6:4 to 9:1 (7). A study covering 20 years of national-scale data (2000–2020) showed that the incidence of MRONJ in patients with breast cancer can be as high as 16% (8).

Current standard treatments for MRONJ are generally divided into conservative and surgical interventions. Conservative therapies, such as antibiotics and adjunctive treatments, are applicable across all stages of the condition, while surgical interventions are typically employed to control the progression of necrosis (2). However, in cases of cancer-related MRONJ, surgical intervention is associated with prolonged hospitalisation and high rate of readmission (9, 10), which contributes to increased morbidity and healthcare burden. Research focusing on MRONJ in the context of cancer therapy remains limited, particularly because there are few models that effectively mimic the combined effects of chemotherapy and immune suppression. Additionally, there is a notable lack of standardised protocols for managing cancer-related MRONJ. Given the rising prevalence of MRONJ, particularly among patients with cancer, there is an urgent need for more effective treatments beyond current standard options.

Considering the limitations of current therapies, drug repurposing, the identification of novel therapeutic applications for existing medications, presents a promising alternative strategy. This approach accelerates drug development by leveraging prior research and established safety profiles, thereby reducing the risks, costs and time constraints typically associated with *de novo* drug development. Repurposing allows for more efficient use of therapeutic compounds and exploration of the untapped potential of various molecules (11, 12).

In 2010, a case series involving six patients demonstrated that the combination of PTX and TOC—the PENTO protocol—promoted healing of necrotic bone in patients with MRONJ (13). Although no results from randomised controlled trials (RCTs) on the PENTO protocol have been published, ongoing trials have been registered (4). Similarly, in 2020, a small RCT involving 34 patients showed that the repurposed use of teriparatide (TPTD), originally approved for osteoporosis, can enhance wound healing and reduce bone loss in patients with MRONJ (14). Moreover, other medications, such as metformin, have shown beneficial effects in osteoporosis rat models treated with zoledronate and dexamethasone, which had delayed jawbone wound healing (15). This suggests the potential for a broader spectrum of repurposed medications.

In this review, we present the current knowledge on systemically administered medications, specifically TPTD and the PENTO protocol, for managing MRONJ. We discuss the mechanisms by which these treatments may prevent or promote the healing of lesions and propose a potential plan for their clinical application. Additionally, based on the proposed mechanisms underlying MRONJ, we outline a broader range of potential repurposed medications, offering relatively safer options, particularly for patients with cancer who are often more vulnerable and face greater limitations in medication selection.

## 2 Mechanisms and medications associated with MRONJ

**Figure 1:**
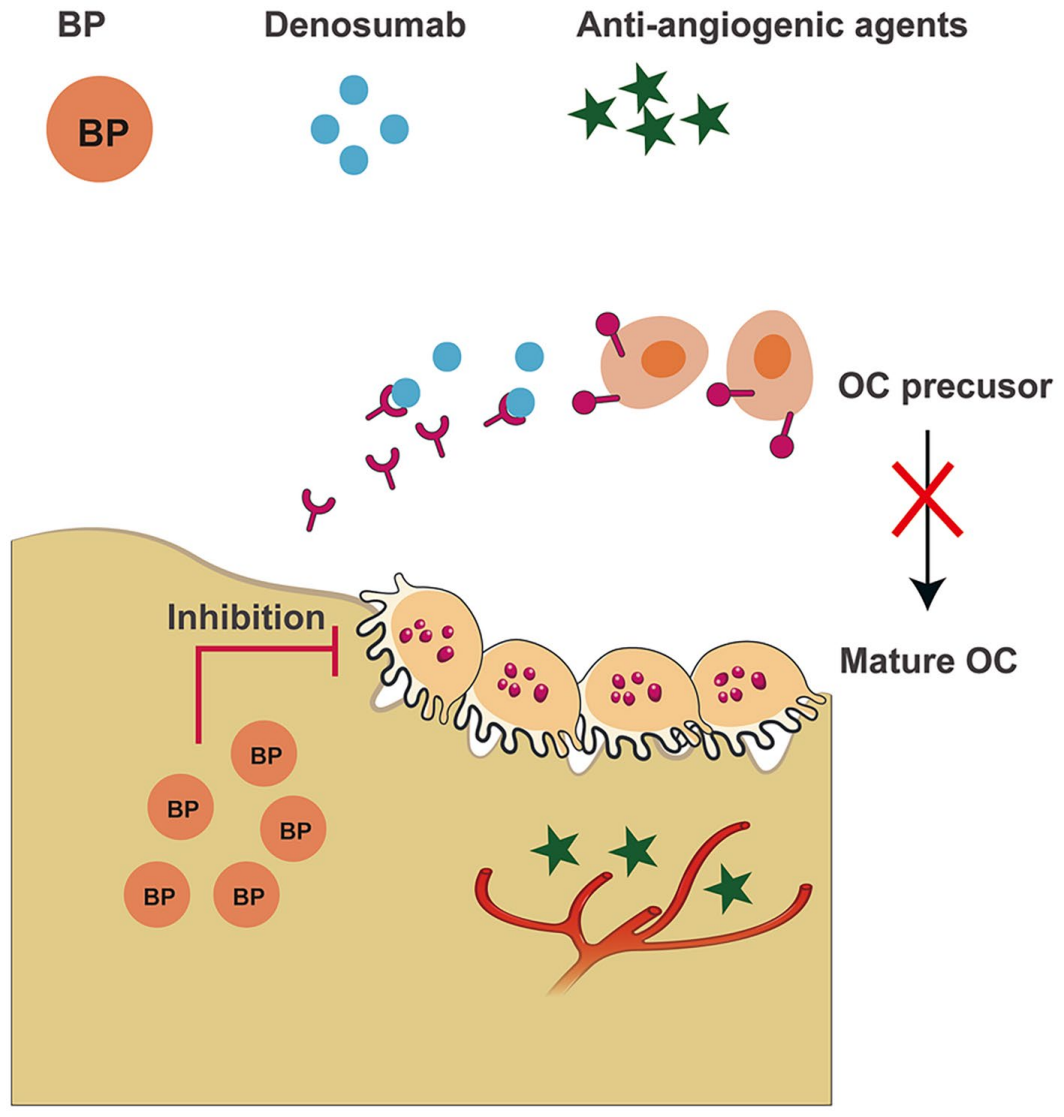
Medications associated with MRONJ. BP can bind to the jawbone for prolonged periods, resulting in sustained inhibition of OC. Denosumab inhibits the differentiation of OC precursors into mature OC by competitively binding to RANKL, and does not exhibit long-term jawbone accumulation. Anti-angiogenic agents reduce blood supply to the jawbone by inhibiting angiogenesis.

Medications associated with the induction of MRONJ can be classified into two primary categories: antiresorptive and antiangiogenic agents, with different mechanisms of action (Figure 1). Antiresorptive agents include BPs and denosumab, both of which inhibit osteoclast (OC) activity, thereby reducing bone resorption. The antiresorptive properties of BPs have led to their widespread use in the management of metabolic bone diseases, such as Paget’s disease, osteogenesis imperfecta, and osteoporosis (16). The use of BPs for the treatment of osteoporosis became widespread in the 1990s, beginning with the introduction of etidronate in the United States (17, 18), followed by the global adoption of alendronate (19–22). Subsequently, additional BPs, including risedronate, ibandronate, pamidronate, tiludronate, and zoledronate, were developed (23–27). The development of new BPs is ongoing with improved versions expected to emerge as efforts continue to meet the growing clinical demand.

**Figure 2:**
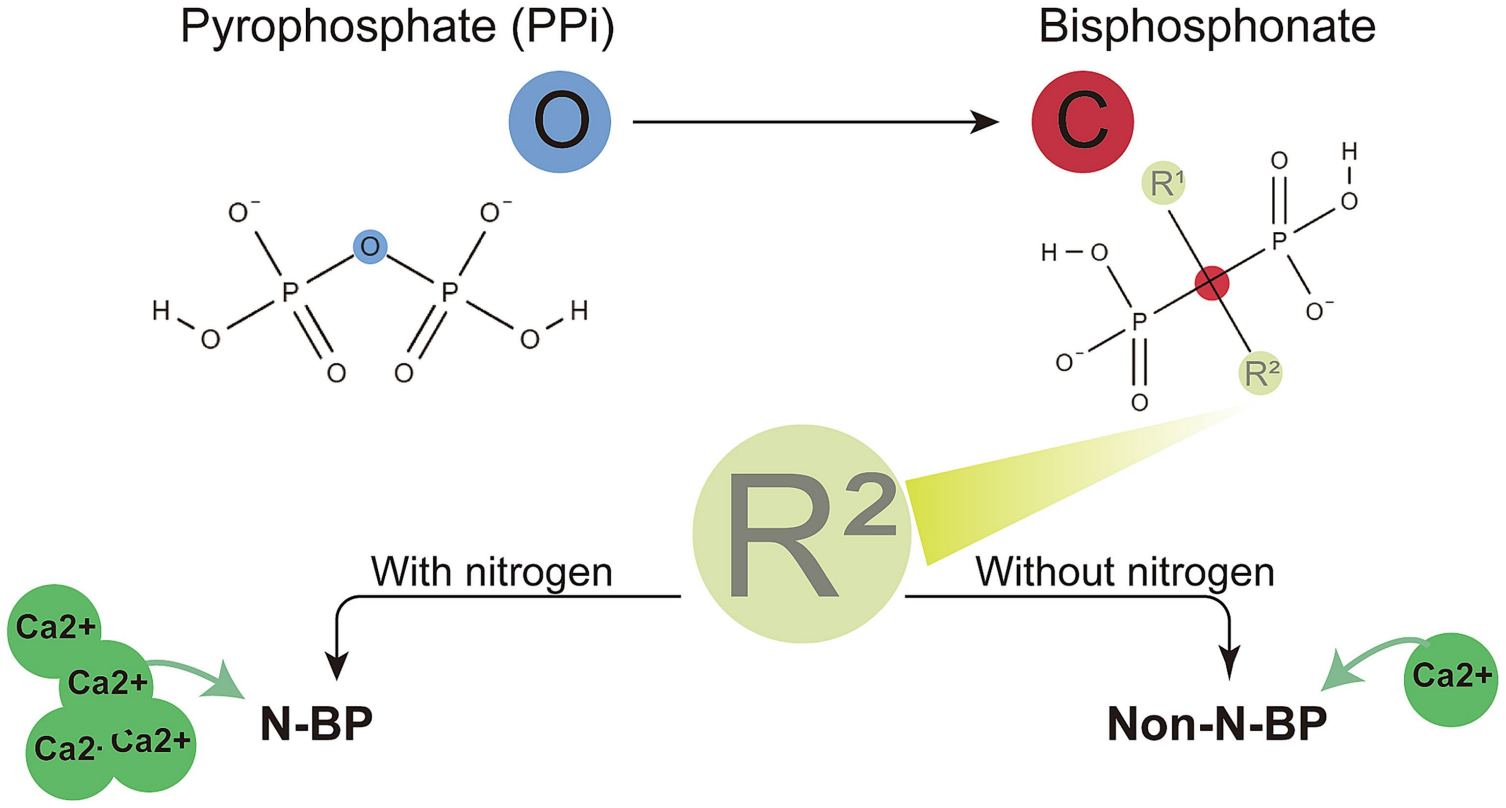
BP structures and types. BPs are classified into two types based on their side chain composition: nitrogen-containing BPs (N–BPs) and non-nitrogen-containing BPs (non-N–BPs). Both N–BPs and non-N–BPs can chelate calcium ions, with N-BPs exhibiting a stronger chelation capacity. Additional medications associated with MRONJ are also depicted.

Structurally, BPs are analogues of inorganic pyrophosphates, with the oxygen atom in the central backbone replaced by a carbon atom. The phosphorus–carbon–phosphorus (P–C–P) backbone structure is connected to two side chains (R_1_ and R_2_). R_1_ is typically a hydroxyl (-OH) group, while R_2_ is often a bulkier group that may or may not contain nitrogen. BPs are classified into nitrogen-containing BPs (N–BPs) or non-nitrogen containing BPs (non-N–BPs) based on the presence of nitrogen in the R_2_ chain. N–BPs inhibit the mevalonate pathway, whereas non-N–BPs are metabolised into non-hydrolysable adenosine triphosphate (ATP) analogues, leading to OC apoptosis (16). Figure 2 shows the BP structure and types.

N–BPs exhibit higher binding affinities than non-N–BPs owing to the formation of strong coordination bonds between nitrogen atoms and metal ions. This enhanced interaction, which is stronger than electrostatic interactions, facilitates the preferential deposition of N–BPs in bone tissues (28, 29). Currently, N–BPs are more widely used in clinical practise than non-N–BPs owing to their superior efficacy (30).

In addition, the receptor activator of nuclear factor kappa-B ligand (RANKL) inhibitor, denosumab, was approved in 2010 for the treatment of bone diseases, and treating cancer metastasis induced skeleton related events (SRE). The same year, denosumab was reported to induce MRONJ (31). Unlike BPs, which bind directly to bone mineral and are internalised by OC, denosumab acts systemically by neutralising RANKL, preventing its interaction with RANK on OC precursors and mature OC. This fundamental difference in mechanism results in distinct pharmacokinetics: denosumab has a shorter half-life (approximately 32 days) and its effects on bone turnover are reversible upon discontinuation (32), whereas BPs exhibit prolonged skeletal retention and persistent suppression of bone remodelling. Clinically, denosumab is administered subcutaneously every 6 months for osteoporosis (60 mg) (33) and every 4 weeks for cancer-related bone metastases (120 mg) (34). The risk of MRONJ appears to be dose- and duration-dependent, with higher incidence observed in oncology patients receiving high-dose, frequent administration compared to osteoporotic patients on standard regimens (2).

Notably, antiangiogenic agents have also been implicated in MRONJ, including the vascular endothelia growth factor (VEGF) inhibitor bevacizumab, approved in 2004 (35); tyrosine-kinase inhibitor sorafenib, approved in 2007 (36); rapamycin target sirolimus, approved in 2008 (37); tyrosine-kinase inhibitor sunitib, approved in 2011; and VEGF inhibitor anlotinib, approved in 2018 (38). The risk of MRONJ with these agents is influenced by treatment duration, cumulative dose, and combination with antiresorptive drugs. In oncology, antiangiogenics are often used at higher doses and for longer periods, thereby increasing MRONJ risk. Furthermore, their concomitant use with BPs or denosumab—common in metastatic cancer management—synergistically disrupts both bone remodelling and vascular homeostasis, exacerbating jawbone vulnerability (38).

The pathophysiology of MRONJ is complex and involves multiple contributing factors, including medication toxicity (39, 40), inhibition of bone remodelling (41–44), impaired angiogenesis (45), trauma (46), infection (47), compromised immune response (48–50), and genetic predisposition (51, 52). Polymorphisms in genes, such as farnesyl pyrophosphate synthase or cytochrome P450 2C8 (CYP2C8), which encodes a cytochrome P450 enzyme, have been identified as risk factors for MRONJ in patients with multiple myeloma (MM) (51, 53). Moreover, other genetic variants, such as VEGF (54), collagen type 1 A 1 (COLIAI) (52), matrix metalloproteinase-9 (MMP9) (55), and peroxisome proliferator-activated receptor gamma (PPARG) (56), have been linked to an increased risk of developing MRONJ. It is likely that multiple factors and pathways are involved in triggering MRONJ.

## 3 Current medications for MRONJ management

### 3.1 TPTD: from osteoporosis to MRONJ

TPTD is a molecule that contains the first 34 amino acids at the N-terminus of the parathyroid hormone (PTH) sequence, also known as PTH (1–34) (57). It was approved by the U. S. Food and Drug Administration (FDA) in 2002 as the first anabolic agent for the treatment of osteoporosis (58). An RCT demonstrated that TPTD can reduce the risk of new vertebral compressions by up to 65% and increase bone mineral density (BMD) by 9% in the lumbar spine (59). Currently, TPTD is administered daily via subcutaneous injection for 18–24 months to lower the risk of vertebral and non-vertebral fractures (60).

However, controversy remains regarding the safety of TPTD, particularly concerning its potential to induce or accelerate cancer. In preclinical studies, TPTD was associated with a dose-dependent increase in the incidence of osteosarcoma in rats (61). However, no cases of osteosarcoma were reported during clinical trials or in a 5-year post-treatment follow-up study, which included seven long-term trials. Only a few spontaneous cases of osteosarcoma have been observed in patients treated with TPTD. Although the osteosarcoma warning was removed from the official drug labelling in 2020, caution is still advised in certain cases. Contraindications for TPTD include a history of radiation therapy, the presence of primary or metastatic bone tumours associated with hypercalcemia, and Paget’s disease (62). Therefore, the use of TPTD is limited in patients with cancer, particularly in those with primary bone tumours, bone metastasis, or a history of radiation therapy.

### 3.2 Efficacy of TPTD in MRONJ treatment: clinical findings

The first reported use of TPTD for the treatment of MRONJ was documented in 2013 (63). In this case report, two patients experienced recovery and regional osteogenesis after 3 months of TPTD injections (administered daily in one case and monthly in the other). More recently, additional case reports and case series have been published. Sim et al. (14) conducted an RCT in which patients with MRONJ received subcutaneous TPTD injections (20 μg/d) or placebos, along with calcium and vitamin D supplementation and standard clinical care, for 8 weeks. The study found that TPTD was associated with a higher rate of MRONJ lesion resolution than the placebo group. Ohbayashi et al. (72) compared daily versus weekly TPTD administration over a 6-month period in patients with osteoporosis and observed significant improvements in MRONJ staging in both the overall patient cohort and daily administration group (14). However, the small sample sizes in both studies (34 and 13 patients) and the distinct disease backgrounds in Sim’s cohort (85.3% of patients with cancer and 14.7% with osteoporosis) limited the generalisability of these findings.

### 3.3 Mechanisms of TPTD action in MRONJ

The mechanism of action of TPTD involves promoting bone remodelling, enhancing osteoblast (OB) activity, and increasing both calcium absorption and reabsorption (64), ultimately leading to increased BMD. However, the precise mechanism by which TPTD restores MRONJ lesion healing remains unclear. Yu et al. (65) hypothesised that activation of the Wnt/β-catenin signalling pathway, promotion of angiogenesis, and downregulation of inflammatory factors play essential roles in the healing of MRONJ. These hypotheses are supported by animal studies (66, 67) on the role of Wnt/β-catenin and by both *in-vitro* (67) and *in-vivo* (68) studies on angiogenesis.

Additionally, rodent models have demonstrated that TPTD can repair osteoarthritis lesions by inhibiting tumour necrosis factor alpha (TNF-α)-mediated upregulation of MMP-13, blocking the infiltration of macrophages and lymphocytes (69), and suppressing the expression of pro-apoptotic and inflammatory molecules, such as Bcl-2-associated X protein (BAX), a disintegrin and metalloproteinase with thrombospondin motifs 5 (ADAMTS5), inducible nitric oxide synthase (iNOS), cyclooxygenase-2 (COX2), interleukin-6 (IL-6), and TNF-α. This suggests a potential role for the regulation of the phosphoinositide 3-kinase/protein kinase B (PI3K/AKT) signalling pathway (70). However, none of these mechanisms have been directly studied in humans.

#### 3.3.1 Clinical protocols for TPTD in MRONJ treatment

Currently, two dosing regimens are recommended for subcutaneous TPTD administration in the treatment of MRONJ: daily (20 μg/d) or weekly (56.5 μg/week). Studies have shown that both dosing schedules are effective and safe (71). The duration of treatment reported in clinical studies varies from 8 weeks (14) to 6 months (72). While no clinical studies have specifically evaluated the use of TPTD for the prevention of MRONJ, rat models indicate that TPTD may reduce the risk of developing MRONJ, suggesting its potential as a preventive approach (73).

### 3.4 PENTO: from ORN to MRONJ

#### 3.4.1 Clinical evidence for the PENTO protocol in MRONJ

In addition to TPTD, recent studies have shown that the pentoxifylline (PTX) and TOC protocol, known as PENTO, can restore necrotic lesion healing in patients with MRONJ (74–76). PTX is a methylxanthine derivative that enhances peripheral blood flow, reduces blood viscosity by increasing vasodilation and erythrocyte flexibility, and inhibits inflammation and fibrosis via its anti-TNF-α properties (77, 78). TOC, a methylated phenol, decreases inflammation, tissue fibrosis, and oxidative stress-induced tissue damage. The combined use of PTX and TOC in the PENTO protocol has demonstrated a synergistic effect in the treatment of MRONJ (79).

The PENTO protocol has also been effective in preventing osteoradionecrosis (ORN) in patients undergoing tooth extractions after head and neck radiotherapy (80). It was first employed in 1997 to treat mucosal injuries following radiotherapy for head and neck cancer (81). Based on the success of treating ORN, PENTO presents as a safer alternative to TPTD, offering suitability for a broader range of patient populations. Since 2010, PENTO has been repurposed to treat MRONJ lesions, although most studies have involved small sample-sizes (13), and it has recently been prescribed to patients for prevention (74).

#### 3.4.2 Hypothesised molecular mechanisms of PENTO in MRONJ treatment

**Figure 3:**
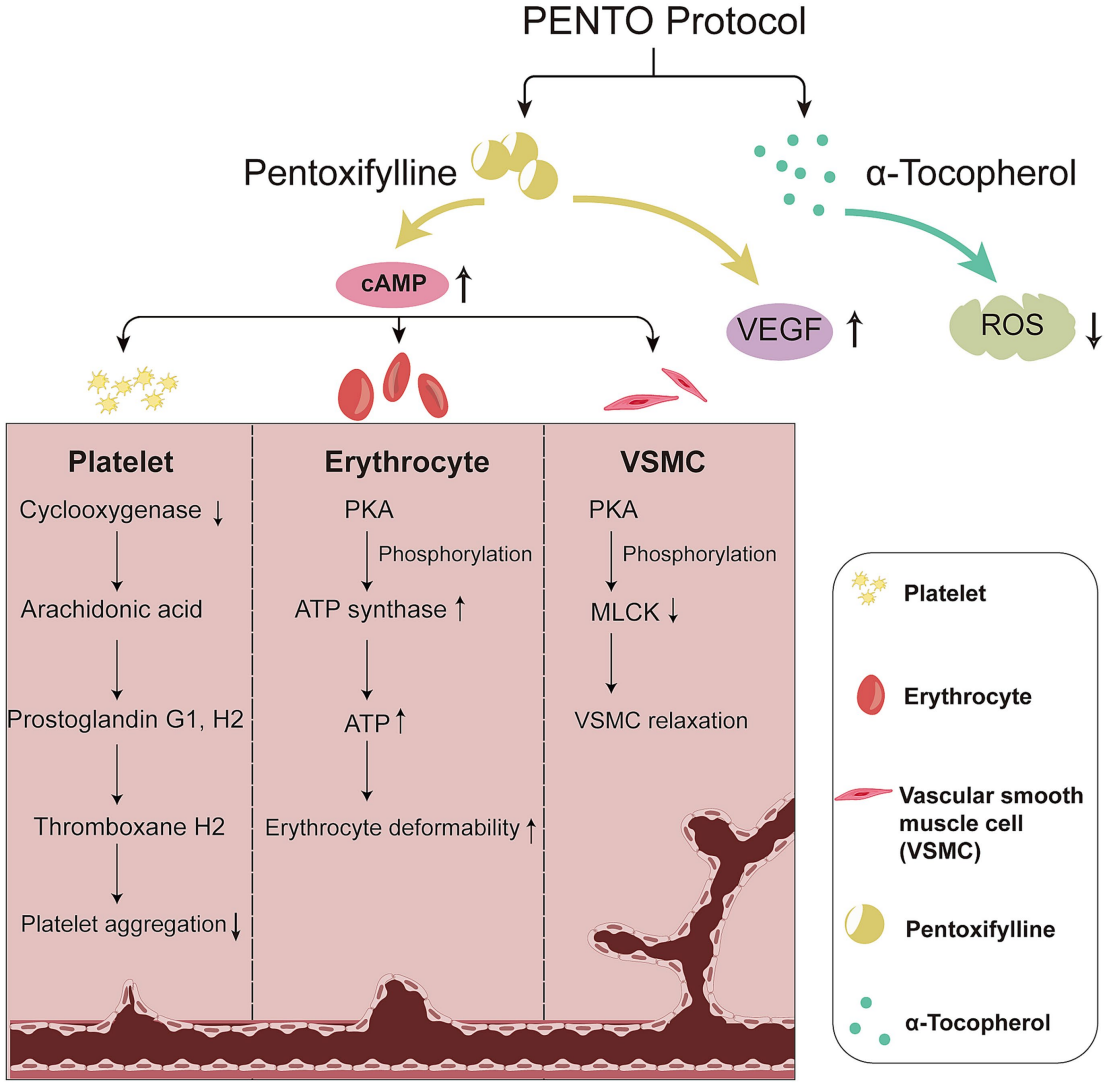
Hypothesis of mechanisms of the PENTO protocol in improving blood supply. To restore blood supply, the PENTO protocol increases cAMP and VEGF levels while reducing oxidative stress. The elevated cAMP level, induced by PTX, triggers a series of effects on platelets, erythrocytes, and VSMC. In platelets, reduced cyclooxygenase levels inhibit the synthesis of arachidonic acid, leading to decreased levels of prostaglandin G_1_ and H_2_, followed by reduced thromboxane A_2_, resulting in reduced platelet aggregation. In erythrocytes and VSMCs, increased cAMP activates the protein kinase A (PKA) pathway, which can elevate ATP levels or reduce MLCK, thereby enhancing the deformability of erythrocytes and the relaxation of VSMCs, ultimately improving blood hemorheology. Additionally, PTX increases VEGF production and TOC presents strong antioxidant effects, contributing to the overall effectiveness of the PENTO protocol.

Herein, we propose a novel hypothesis for the treatment of MRONJ using the PENTO protocol (Figure 3). We hypothesised that PTX, a nonspecific phosphodiesterase (PDE) inhibitor, blocks the hydrolysis of cyclic adenosine monophosphate (cAMP), triggering a series of biological interactions that restore impaired regional blood supply and promote bone remodelling in MRONJ. TOC, by mitigating oxidative tissue damage, further enhances the therapeutic outcome. The combined use of PTX and TOC is expected to exert a synergistic effect owing to their complementary pathways. This hypothesis presents a new approach to managing MRONJ and could lead to the development of more targeted preventive and therapeutic strategies.

##### 3.4.2.1 PENTO and blood supply restoration in MRONJ

MRONJ is typically characterised by avascular necrosis, where the breakdown of the capillary network supplying blood to the bone leads to necrosis. We hypothesised that the PENTO protocol, particularly through the enhancement of cAMP by PTX, contributes to restoring these essential capillary networks.

Studies have shown that BPs can reduce arterial and venous areas and overall periodontal vascularity in rat models (82, 83). We hypothesised that PTX, by increasing cAMP levels, may promote microvascular growth and stabilisation, thereby aiding in the restoration of MRONJ lesions. One of the key challenges in MRONJ treatment is accurately determining the disease margins, as microvascular abnormalities in the mucosa near MRONJ lesions can complicate diagnosis (84).

Animal studies have shown a reduction in the number of blood vessels and microvessels at the extraction socket (85) within MRONJ sites. Given the essential role of angiogenesis in maintaining adequate blood supply, the antiangiogenic properties of BPs are significant. BPs have been shown to inhibit angiogenesis both *in vitro* and *in vivo* (45, 86, 87), leading to reduced vascularity and impaired healing following tooth extraction. Therefore, restoring regional blood flow and promoting angiogenesis are critical for effective MRONJ management.

PTX enhances blood circulation by improving red blood cell deformability, reducing blood viscosity, and lowering plasma fibrinogen levels (88). It also inhibits platelet aggregation by blocking membrane-bound PDE, increasing cAMP levels, and promoting prostacyclin synthesis while inhibiting thromboxane production (77, 78). These effects lead to improved regional blood supply, which can support the restoration of avascular necrosis in the jawbone.

In MRONJ lesions, angiogenesis is typically impaired. Although PTX has been associated with antiangiogenic effects in tumour models (89, 90), recent studies indicate that it can also improve insufficient angiogenesis in bone tissue (91, 92). PTX angiogenic effects are thought to be mediated by increased levels of VEGF (91), and these effects may be dose- and duration-dependent (93). Furthermore, we hypothesised that the antioxidant action of PTX, when combined with TOC, could enhance vascular endothelial repair and reduce oxidative stress-induced vascular damage (94).

Similarly, TOC administration leads to a biphasic modulation of VEGF. TOC can reduce elevated VEGF levels in cancer cells (95, 96), but it restores impaired VEGF expression under conditions of high glucose and excessive oxidative stress, possibly through the hypoxia-inducible factor (HIF) pathway (97). A similar effect has been observed in the placental vascular network of late-pregnant ewes (98). Furthermore, TOC enhances the clearance of free radicals (99), further supporting the use of the PENTO protocol to restore blood supply.

In summary, the PENTO protocol may improve blood supply through the hemorheological effects of PTX, with PDE inhibition and cAMP elevation playing key roles in this process. The restoration of VEGF-mediated angiogenesis further strengthens the therapeutic potential of this approach.

##### 3.4.2.2 PENTO and restoration of bone remodelling in MRONJ

Inhibition of bone remodelling is a key factor in the development of MRONJ (2). Antiresorptive agents commonly implicated in MRONJ, such as BPs and denosumab, suppress bone resorption through distinct mechanisms: BPs induce OC apoptosis (16) whereas denosumab inhibits OC formation by blocking the receptor activator of RANKL (100). Consistent with this, animal studies and clinical observations have demonstrated the presence of dysfunctional or absent OCs in denosumab-treated mice and BP-treated patients (101). Although discontinuation of denosumab prior to tooth extraction can prevent MRONJ development in rat models (102), established lesions generally fail to resolve following withdrawal of antiresorptive therapy. On this basis, we propose that the dual action of PTX and TOC within the PENTO protocol may help counteract these pathological effects by restoring both OB and OC activity.

The bone remodelling pathway, which comprises transforming growth factor beta (TGF-β)1 signalling, has been identified as a contributing factor in MRONJ pathogenesis (103). TGF-β1 also influences the RANKL/osteoprotegerin (OPG) signalling pathway, which affects osteoclastogenesis and bone turnover. Furthermore, PTH, which impacts bone formation and remodelling, can prevent MRONJ and enhance post-extraction healing (104), underscoring the importance of OC inhibition in MRONJ development. These factors collectively highlight the intricate relationship between bone remodelling impairment and MRONJ pathogenesis.

**Figure 4:**
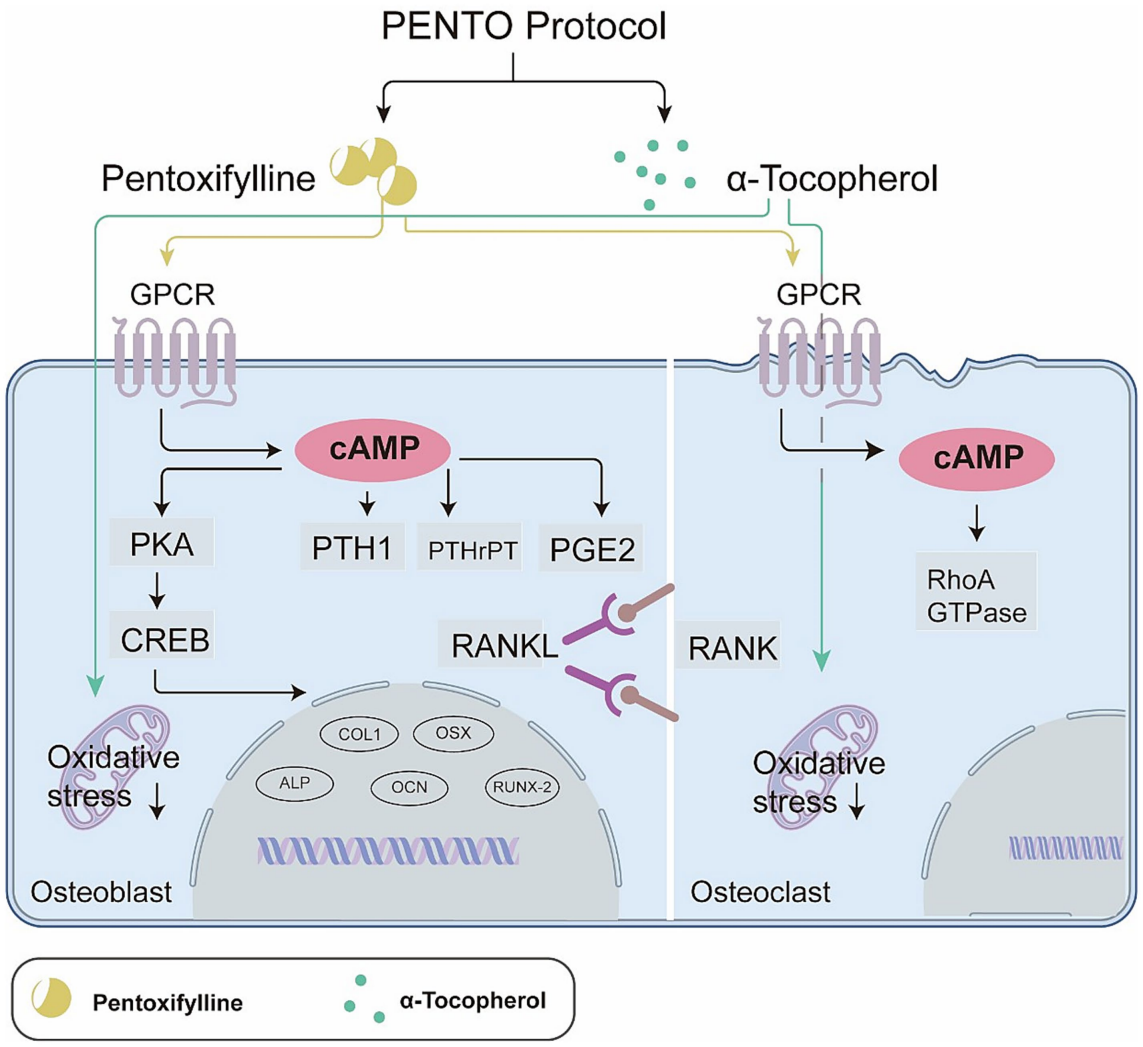
Regulatory effects of the PENTO protocol on OB and OC. Both OB and OCs are restored by the PENTO protocol. In OB, the increased cAMP levels PKA-cAMP-CREB pathway. This, along with the effects of PTH1, PTHrP, and PGE_2_, promotes osteogenic differentiation, resulting in elevated RANKL levels and increased OC differentiation. In OC, increased RhoA-GTPase enhances the mobility of OCs, thereby increasing resorption activity. Furthermore, the reduction in oxidative stress in both osteoblasts and OCs promotes cellular activity.

As a nonselective PDE inhibitor, PTX increases intracellular cAMP levels, promoting mesenchymal stem cell proliferation and differentiation into OB (105). cAMP activates protein kinase A (PKA), which phosphorylates the cAMP response element-binding protein (CREB), triggering the transcription of osteogenic genes. However, sustained cAMP signalling inhibits OB differentiation and mineralisation (106). This dual effect of PTX is mediated by the cAMP–PKA–cAMP CREB pathway. Their effects on OB are exerted through their respective G protein-coupled receptors (GPCRs), which activate membrane-bound adenylyl cyclase (AC) to convert ATP to cAMP. Additionally, cAMP production in endosomes is dependent on β-arrestin-1/2 and is sustained by the extracellular signal-regulated kinase 1/2 (ERK1/2) pathway. This noncanonical endosomal signalling pathway is terminated by a negative feedback loop, which is mediated by PKA-dependent vacuolar-ATPase phosphorylation. While the cAMP–PKA–CREB pathway directly stimulates RANKL levels in OB, hormones, such as PTH, parathyroid hormone-related peptide (PTHrP), and prostaglandin E2 (PGE2), can also promote OC formation by increasing RANKL production through this pathway. Additionally, cAMP can activate the guanosine triphosphate hydrolase (GTPase) ras homologue gene family member A (RhoA), which is essential for OC movement (Figure 4).

Furthermore, the role of TOC in reducing proinflammatory cytokines and reactive oxygen species (ROS) might be critical in preserving bone integrity and promoting bone healing in patients with MRONJ. The potential of TOC to promote bone remodelling has gained considerable attention (64) owing to its bone-protective effects, which are primarily mediated through the suppression of antiosteogenic factors and bone resorption. TOC’s primary mechanism lies in its ability to reduce ROS levels and various proinflammatory cytokines, including interleukin (IL)-1, IL-6, PGE2, and TNF-α, all of which are known to induce OC activity. By inhibiting OC activity, TOC contributes to the preservation of bone mass.

A study conducted by Muhammad et al. (107) using animal models demonstrated that TOC and tocotrienol effectively reduced bone resorption and bone loss. Furthermore, observations in postmenopausal women have revealed an association between low serum α-TOC levels and vitamin E intake, in addition to increased risk of osteoporosis and hip fracture, highlighting the importance of TOC in maintaining bone health (108, 109). Interestingly, TOC supplementation has been shown to accelerate orthodontic tooth movement by increasing RANKL expression and promoting alveolar OC proliferation, although this effect is not observed in long bones (110). While the precise mechanism underlying these effects remains unclear, the potential of TOC to modulate bone metabolism continues to attract research interest. Notably, PTX and TOC have been shown to promote bone remodelling through distinct pathways, suggesting a synergistic effect when used in combination.

##### 3.4.2.3 Strength of evidence and clinical relevance of proposed mechanisms

Overall, among the proposed mechanisms, those related to enhanced bone remodelling and improved microcirculation are supported by the most consistent clinical and translational evidence. In particular, TPTD-associated promotion of OB activity and bone turnover has been demonstrated in both clinical MRONJ studies and controlled trials, while the hemorheological and vasodilatory effects of PTX, together with the antioxidant properties of TOC, are supported by clinical observations of improved soft tissue healing and symptom resolution. In contrast, several intracellular signalling pathways described in this review, including specific cAMP–PKA–CREB cascades, RhoA-mediated OC movement, and VEGF upregulation, are primarily derived from *in vitro* or non-MRONJ models and should therefore be considered mechanistic hypotheses rather than clinically validated pathways. Further well-designed translational and clinical studies are required to clarify the relative contribution of these molecular mechanisms to therapeutic outcomes in MRONJ.

#### 3.4.3 Protocol for clinical applications

According to completed studies, the PTX and TOC dosage is 400 mg twice a day or 800 mg once daily for treatment, and 400 mg daily for prevention, administered orally. The prescription duration depends on whether the protocol is combined with surgery. For the single-use protocol, treatment duration ranges from 2 to 7 months (111) and can be extended up to 6 months (76). When combined with surgery, 3 months perioperatively is suggested (112). For patients without existing lesions who require dental extraction, pre- and post-surgery PENTO administration for 2 weeks is recommended (113).

Generally, PENTO should be prescribed both before and after surgery when combined with surgical intervention. In cases of standalone treatment without surgery, a longer duration is expected. Dosage and duration may be reduced for preventive use. However, given the variability in systemic conditions among patients, practical dosage and treatment duration should be tailored on a case-by-case basis.

### 3.5 TPTD vs. PENTO

TPTD and PENTO both offer promising potential in the treatment and prevention of MRONJ. A comparison of their possible targeting pathways, administration routes, dosages, durations, adverse effects and limitations is summarised in Table 1. TPTD, originally repurposed from treating osteoporosis, targets the Wnt/β-catenin and angiogenesis pathways via multiple related factors. It also has anti-inflammatory effects, reducing levels of IL-1β, TNF-α, IL-6, iNOS, and COX-2. TPTD is administered either daily or weekly, via subcutaneous injection. However, owing to its potential carcinogenic risk, TPTD should not be used in patients with cancer. In addition, hypercalcaemia, dizziness and nausea have been reported as adverse effects of TPTD (114).

**Table 1:** TPTD vs. PENTO: targets, administration, dosing, safety, and limitations.

Comparisons	TPTD	PENTO
Possible targeting pathways	Wnt/β-catenin (osteogenesis), angiogenesis, anti-inflammation	PTX: Blood supply (microvascular permeability, RBC deformability, vasodilatory, ATP supply). TOC: Antioxidant
Possible factors	TCF proteins, VEGF, Ang-1, CD31, IL-1β, TNF-α, IL-6, iNOS and COX-2	COX-1, PKA, VEGF, ROS, PTH1, PTHrP, PGE2, and RhoA-GTPase
Previously used for	Osteoporosis	ORN
Treatment/prevention	Treatment	Treatment and prevention
Adaption MRONJ	Osteoporosis	Osteoporosis and cancer
Administration	Subcutaneous injection	Oral
Dosage	20 μg/d or 56.5 μg/week	Treatment: 400/600 mg BID or 800 mg QD for PTX, 400 mg BID or 800 mg QD for TOC. Prevention: 400 mg QD for PTX and TOC
Duration	6–8 weeks	Treatment: 3–17 months. Prevention: 2 weeks perioperatively
Adverse effects	Potential osteosarcoma risk, hypercalcaemia, dizziness and nausea	PTX: gastrointestinal discomfort, dizziness, headache and rare bleeding risk TOC: generally well tolerated; high-dose or long-term use may increase bleeding tendency
Limitations	limited applicability in cancer-associated MRONJ	Limited concurrent use with potent anti-angiogenic agents due to the risk of bleeding

In contrast, the PENTO protocol, repurposed from ORN treatment, is used for both treating and preventing MRONJ. PENTO affects pathways related to microvascular permeability, red blood cells deformability, vasodilation, ATP supply, and antioxidant activity. PENTO is typically administered orally, and the dosage and duration for prevention MRONJ are significantly lower than those for treatment. Unlike TPTD, PENTO has no reported carcinogenic effect and even shows anti-cancer effects on multiple cancer types (115, 116), making it suitable for a broader patient population, including patients with cancer. For PENTO, common adverse effects include gastrointestinal upset, dizziness, and headaches for PTX, while high doses of TOC can cause nausea or diarrhoea. Overall, PENTO has been reported to be safe for patients with ORN and poses no carcinogenic risk.

### 3.6 Clinical considerations for cancer patients

TPTD exerts anabolic effects through activation of PTH and downstream signalling pathways involved in bone remodelling, angiogenesis, and cellular proliferation (62), which may theoretically influence tumour–bone interactions. However, the initial rat studies utilised doses 3–60 times higher than.

therapeutic levels for near-lifetime exposure—conditions that do not reflect clinical practise (61). In addition, currently no mechanistic studies have successfully replicated osteosarcomagenic effects in human cell lines or more relevant models.

Unlike TPTD, PENTO improves MRONJ lesion through indirect mechanisms without altering bone metabolism or the tumour-bone microenvironment, thus avoiding theoretical risks in oncologic populations. However, it also faces limitations in cancer polypharmacy. The major constraint is bleeding risk in anticoagulated patients—prevalent in oncology due to thromboprophylaxis needs—requiring INR monitoring (117) and may rarely associated with thrombocytopenia (118). PTX’s hepatic metabolism may be impaired in patients with liver metastases, therefore worsen fatty liver in patients with pre-existing hyperglycaemia (61). For TOC, there are also concerns about the attenuated chemotherapy or radiotherapy efficacay raised by antioxidatnt (119, 120), though clinical evidence is lacking. These factors necessitate careful patient selection and enhanced monitoring, potentially limiting PENTO’s applicability in heavily pretreated or medically complex oncologic populations.

## 4 Repurposing other medications for MRONJ

Traditional drug discovery is a complex and time-consuming process that involves multiple stages. The drug development process takes an average of 10 years and approximately 1 billion US dollars in costs, with a success rate of only 2.0% (121–123). Drugs that fail in phases 2 and 3 owing to poor efficacy, but with no safety-related issues in the intended indication, are often withdrawn from the market. Currently, there are more than 7,000 rare and neglected diseases lacking effective therapies, or facing issues, such as drug resistance (124, 125). Given the high costs and long timelines of traditional drug discovery, repurposing existing drugs for new indications, including cancer and rare diseases, is gaining traction. This approach uses drugs that have already passed essential development phases, offering potential treatments for patients with limited options while saving both time and money.

Drug repurposing strategies can be divided into three categories: drug-centric, disease-centric, and target-centric, depending on the screening methods used (126). In the context of MRONJ, drug-centric approaches focus on treatments like TPTD and PENTO, disease-centric approaches target osteonecrosis (e.g., osteoradionecrosis [ORN] or femoral head necrosis [FHN]), and target-centric approaches predict new treatments based on current known pathways.

### 4.1 Approaches used for repurposing

#### 4.1.1 Drug-centric approaches

Potential medications for MRONJ can be identified based on the mechanisms of MRONJ and pharmacological targets of TPTD and PENTO. Given the differences between osteoporosis and patients with cancer, treatment options can be categorised accordingly. For osteoporosis-related MRONJ, drugs similar to TPTD can be considered, while non-carcinogenic alternatives are preferable for patients with cancer.

There are two drug-centric approaches: identifying drugs with structures similar to the primary drug or identifying drugs that perform similar physiological functions. For example, abaloparatide (ABL), another PTH analogue, is structurally similar to TPTD and is prescribed for osteoporosis without causing MRONJ (127). ABL is currently in clinical trials, with a review of RCTs showing that it improves BMD in postmenopausal women with osteoporosis more effectively than TPTD (128). As ABL targets the same pathways as TPTD, it may be a potential treatment option for MRONJ.

For PENTO, other methylxanthines like caffeine, theophylline, theobromine, and propentofylline have chemical structures similar to PTX. However, tocotrienols and α-tocopheryl acetate are more closely related to TOC. Replacing PTX with single methylxanthines may lead to unpredictable outcomes (129), making them less reliable as alternatives. Cilostazol, which improves blood flow and reduces vascular viscosity like PTX, has been used to treat intermittent claudication and may be a suitable option (130). Additionally, antioxidants, such as ascorbic acid (131) and coenzyme Q10 (CoQ10) (132), share antioxidant properties with TOC, offering potential alternatives for patients with cancer. However, clinical trials are necessary before repurposing any of these drugs for MRONJ.

#### 4.1.2 Disease-centric approaches

A disease-centric approach identifies candidate drugs based on shared pathological mechanisms between diseases, allowing therapies effective in related conditions to be considered for MRONJ. As PENTO was originally used to treat ORN, other treatments for ORN may be candidates for MRONJ as well. Currently, no medications are specifically approved for ORN treatment. In avascular osteonecrosis, femoral head necrosis (FHN) is the most common condition leading to total hip arthroplasty (THA) in young adults (133). Treatments, such as enoxaparin, statins, BPs, iloprost, and acetylsalicylic acid, have shown potential benefits. However, a review of clinical studies concluded that the evidence is insufficient to make strong recommendations for these treatments (134).

#### 4.1.3 Target-centric approaches

Candidate drugs for MRONJ can be identified by targeting relevant pathways, with a focus on those that promote bone remodelling and angiogenesis. Antioxidants and antibiotics may provide additional benefits. Advanced technologies, such as gene array, protein–protein interaction (PPI) networks, artificial intelligence (AI), and machine learning, can further refine drug screening processes and lead to more precise treatment options.

## 5 Clinical trials for MRONJ

Despite the lack of effective and safe management strategies for MRONJ, various experimental studies are underway. The safety and efficacy of potential therapies must be evaluated and confirmed in clinical phase studies. The US National Institutes of Health (NIH) defines clinical trials as research studies involving human volunteers assigned to protocol-based interventions and assessed for their effects on biomedical or health outcomes (135).

### 5.1 Growth factors and surgical interventions

High quality clinical trials for MRONJ are limited. A review by Beth-Tasdogan et al. (4) analysed 1,047 studies focused on MRONJ management, including 13 RCTs in the final analysis (other studies were excluded owing to the high risks of bias). Among the completed studies, five investigated preventive strategies and eight focused on treatment.

Among the 13 trials, 4 used products rich in growth factors, including plasma rich in growth factors (PRGF), platelet rich fibrin (PRF), and concentrated growth factor (CGF), to prevent or treat MRONJ. These products are derived from the patient’s own blood through specific centrifugation processes and are endowed with the capacity to enhance tissue regeneration and repair. This approach has applications in fields, such as oral surgery, orthopaedics, and dermatological regenerative therapies (136, 137).

Surgical interventions have been explored in several studies. Two trials used autofluorescence to guide jawbone resection, while another compared different surgical protocols for post-extraction wound closure. These advances in surgical techniques aim to minimise pharmacological risks and trauma, but they have not significantly improved MRONJ outcomes.

### 5.2 Pharmacological trials

Among the 13 trials, 2 investigated TPTD injections for treating MRONJ, yielding promising results. Sim et al. administered subcutaneous TPTD injections (20 mg/d) or placebos, along with calcium and vitamin D supplementation, to patients with MRONJ over 8 weeks. Their findings showed a higher rate of lesion resolution in the TPTD group than that in the placebo group (14). Ohbayashi et al. compared daily and weekly TPTD administration over 6 months in patients with osteoporosis, observing notable improvements in MRONJ staging in both the entire cohort and daily group (72). However, the small sample sizes (34 and 13 patients, respectively) and differences in patient populations (Sim’s cohort included 85.3% patients with cancer and 14.7% patients with osteoporosis) limit the reliability of these findings.

Additionally, as mentioned above, TPTD was associated with an increased risk of osteosarcoma in a phase III study (138), posing significant risks for patients with cancer and contraindicating its use in patients with pre-existing hypercalcemia, severe renal impairment, metabolic bone diseases (e.g., hyperparathyroidism or Paget’s disease), unexplained elevated alkaline phosphatase, and prior radiation therapy to the skeleton (139). As a result, TPTD use in patients with MRONJ has been greatly restricted.

Two ongoing RCTs are currently examining the effects of the PENTO protocol in patients with MRONJ. Recent studies have shown that patients with MRONJ and osteoporosis who received PENTO experienced faster healing after tooth extraction and a lower recurrence rate than controls (112). Unlike TPTD, PENTO does not carry the risk of osteosarcoma or disruption of bone metabolism, making it a viable option for a wider range of patients.

Of the 13 RCTs (140, 141), 2 indicated that applying autologous platelet concentration (APC) to the tooth socket led to lower MRONJ occurrence than that under the standard treatment. Additionally, 3 of the 13 RCTs (142–144) combined APC with surgical interventions, resulting in a higher proportion of mucosal coverage than that of surgical intervention alone. However, the small sample sizes may have affected the ability to detect significant effects.

Overall, methodological constraints of the trials were associated with a high risk of bias, contributing to uncertainty about any estimates of effect (4). While various strategies have been proposed to prevent or treat MRONJ, there is insufficient evidence to confidently claim the effectiveness of any tested interventions. Identifying additional drugs for repurposing may offer safer and more effective treatment options in the future.

### 5.3 Critical appraisal of clinical evidence

Table 2 shows the published clinical studies for TPTD (*n* = 12) and PENTO (*n* = 9) in MRONJ management. Both TPTD and PENTO showed potential benefits, but the overall level of evidence remains low. Only 6 out 12 TPTD studies compared with control group, while 1 of 9 PENTO studies did the comparison. Most TPTD data derive from small case reports/series and retrospective cohorts, with only one very small RCT (Sim et al., *n* = 15) (14), while PENTO is supported by a heterogeneous mix of case series, retrospective cohorts and one large RCT (Colapinto et al., *n* = 202, stage I MRONJ in osteoporotic patients) (112).

**Table 2:** Existing clinical studies using TPTD or PENTO for MRONJ management.

Study	Intervention	Combined with surgical intervention	Study design	Prevention or treatment	Sample size	Patient population	BMA history	BMA during treatment	MRONJ stage	Treatment duration	Primary outcome	Main results	Compare to control
Singh et al. (166)	TPTD	Yes	Case report	Treatment	1	Cancer	Denosumab	Not specified	2	6 months	MRONJ resolution	100%	N/A
Park et al. (145)	TPTD	Partially	Retrospective study	Treatment	76	Osteoporosis	BP or denosumab	Altered/stopped	1–3	3–6 months	Healing period	Reduced healing period	Yes
Kim et al. (167)	TPTD	Yes	Case report	Treatment	2	Osteoporosis	BP and/or denosumab	Not specified	3	8–16 months	MRONJ resolution	100%	N/A
Kim et al. (149)	TPTD	Yes	Case control study	Treatment	29	Osteoporosis	BP and/or denosumab	Not specified	1–3	6.4–24.8 weeks	MRONJ resolution	Daily: 88.2%; weekly: 75%	Yes
Choi et al. (168)	TPTD	Yes	Case report	Treatment	3	Osteoporosis	BP and/or denosumab	Altered/stopped	2–3	4–15 months	MRONJ resolution	100%	N/A
Ohbayashi et al. (72)	TPTD	Mostly yes	Pilot study	Treatment	12	Osteoporosis	BP	Not specified	2–3	6 months	MRONJ stage improvement	Daily: 83.3%; weekly: 50%	Yes
Morishita et al. (146)	TPTD	Mostly yes	Retrospective study	Treatment	29	Not specified	BP and/or denosumab	Not specified	1–3	0.3–26 months	MRONJ resolution	75.9%	N/A
Sim et al. (14)	TPTD	Partially	RCT	Treatment	15	Cancer and osteoporosis (*n* = 12, 3)	BP and/or denosumab	Altered/stopped	0–3	2 months	MRONJ resolution	45.4%	Yes
Kakehashi et al. (147)	TPTD	Yes	Case series	Treatment	8	Osteoporosis	BP	Yes	2–3	4–24 months	MRONJ resolution	87.5%	N/A
Kim et al. (148)	TPTD	Yes	Retrospective study	Treatment	15	Osteoporosis	BP	Not specified	2–3	6 months	MRONJ stage improvement	37.5%	Yes
Pelaz et al. (169)	TPTD	No	Pilot study	Treatment	4	Osteoporosis	BP	Not specified	3	4–10 months	MRONJ resolution	25%	No
Kwon et al. (170)	TPTD	Mostly yes	Case series	Treatment	6	Osteoporosis	BP	No	2–3	1–3 months	MRONJ resolution	100%	N/A
Słowik et al. (171)	Pento	No	Prospective study	Treatment	43	Cancer and osteoporosis (*n* = 33, 10)	BP	No	1–3	12 months	MRONJ stage improvement	46%	N/A
Faverani et al. (172)	Pento	No	Case series	Treatment	14	Osteoporosis, osteopenia, cancer, multiple myeloma, rheumatoid arthritis and osteoblastoma (*n* = 4, 3, 3, 2, 1, and 1)	BP	Mostly still on	Not specified	Not specified	MRONJ stage improvement	Not specified	N/A
Colapinto et al. (112)	Pento	Yes	RCT	Treatment	202	Osteoporosis	BP	Yes	1	8 months	MRONJ resolution	100%	Yes
Magalhães et al. (74)	Pento	Yes	Case series	Prevention	17	Cancer	BP	Yes	At risk	3 months	MRONJ occurrence after tooth extraction	17.6%	N/A
Varoni et al. (173)	Pento	No	Retrospective study	Treatment	35	Osteoporosis, cancer and multiple myeloma (14, 14, and 7)	BP and/or denosumab	Many had discontinued	1–3	3.8 months	MRONJ resolution	At 1 month: complete healing 88.57%; At 3 months: complete healing 92%	N/A
Rivas et al. (174)	Pento	No	Case report	Treatment	1	Cancer	BP	No	Not specified	9 months	MRONJ resolution	100%	N/A
Seo et al. (111)	Pento	Yes	Retrospective study	Treatment	9	Osteoporosis and multiple myeloma (*n* = 8, 1)	BP	Not specified	2–3	3–6 months	Radiographic bone density/bone healing trend	Not specified	N/A
Owosho et al. (76)	Pento	No	Case series	Treatment	7	Cancer and multiple myeloma	BP	Mostly no	0–3	3–48 months	MRONJ resolution	Symptom relief and radiographic new bone fill in all; some but not all sites fully healed	N/A
Magremanne et al. (175)	Pento	No	Case report	Treatment	1	Osteoporosis	BP	No	3	12 months	MRONJ resolution	100%	N/A

In treatment settings, reported complete resolution or stage-improvement rates with TPTD are generally high (145–148) and daily TPTD appears more effective than weekly dosing in limited comparative data (72, 149). PENTO likewise shows favourable outcomes in several non-surgical cohorts and, in Colapinto’s trial, pre-operative PENTO plus standard surgery achieved 100% clinical and radiographic healing and clearly outperformed surgery alone in early-stage osteoporotic MRONJ (112). However, prevention data are extremely limited: in the only PENTO prophylaxis series (74), 17.6% of high-risk cancer patients still developed MRONJ after tooth extraction, and there was no comparison with a control group, indicating that any preventive effect is unproven.

Critically, the evidence base is characterised by marked heterogeneity and substantial risk of bias. Sample sizes are small, most studies are single-arm and non-randomised. Patient populations are mixed (osteoporosis vs. cancer; BP vs. denosumab), BMA exposure (dose, route, duration) is poorly characterised, and BMA management during treatment (continued, altered, or stopped) varies widely. The extent of concomitant surgical intervention also differs substantially between and within studies, making it difficult to disentangle drug effects from surgical effects. Outcomes are often clinician-defined “healing” or stage change, with limited use of standardised radiographic or patient-reported endpoints. Taken together, current clinical data suggest that both TPTD and PENTO may be useful adjuncts, particularly in osteoporotic and early-stage MRONJ, but they are insufficient to define standard indications, optimal regimens, or comparative effectiveness. Larger, well-designed randomised trials with rigorous stratification (cancer vs. osteoporosis, high- vs. low-dose BMA, surgical vs. non-surgical) and standardised outcome measures are urgently needed.

### 5.4 Comparison of clinical evidence and guidelines

Consistently, across existing guidelines and consensus statements, TPTD and PENTO are generally regarded as potential or experimental options rather than established MRONJ therapies. The current guidelines and consensus are listed in Table 3. Several position papers from North America, Europe (150), Italy and Belgium (151, 152), as well as the 2025 Korean multidisciplinary task force statement (153) and the North American/European endocrine taskforce (2, 154), mention TPTD but uniformly emphasise that current evidence is limited and further studies are required before routine use can be recommended. In contrast, two documents provide more concrete guidance in selected non-cancer populations: the Colombian multidisciplinary consensus (2020) (154) and the Chinese expert consensus (2024) (155) explicitly suggest TPTD for MRONJ in patients without malignancy, recommending 20 μg subcutaneously once daily, for 24 months and at least 8 weeks, respectively. The Chinese consensus is also the only one to specify a PENTO protocol, listing PTX 400 mg twice daily combined with TOC 400–500 IU twice daily. Overall, these recommendations highlight a cautious, case-selected use of TPTD and PENTO, reserved mainly for refractory MRONJ in non-oncologic patients, within a context of still-insufficient high-quality evidence.

**Table 3:** Guidelines and consensus mentioned TPTD or PENTO for MRONJ management.

Year	Country/Reg	Society	Patient population	Title	Opinion to TPTD	Recommend TPTD dosage	Opinion to PENTO	Recommend PENTO dosage
2025 (153)	Korea	Multidisciplinary task force	Osteoporosis	Medication-Related Osteonecrosis of the Jaw: An Evidence-Based 2025 Position Statement from a Korean Multidisciplinary Task Force	Listed as an option in bone- metabolism regimens for sequential therapy/reducing rebound fracture risk	N/A	N/A	N/A
2025 (150)	North America and Europe	Endocrine practise	Osteoporosis	Antiresorptive Therapy to Reduce Fracture Risk and Effects on Dental Implant Outcomes in Patients With Osteoporosis: A Systematic Review and Osteonecrosis of the Jaw Taskforce Consensus Statement	Mentioned but need more evidence	N/A	N/A	N/A
2024 (155)	China	Oncology and OMFS societies	All MRONJ	Chinese expert consensus on the diagnosis and clinical management of medication-related osteonecrosis of the jaw	The management of MRONJ in patients in the non-cancer setting	Subcutaneous for at least 8 weeks	Listed	PTX 400 mg, bid, and TOC 400–500 IU, bid
2024 (151)	Belgium	Oncology and OMFS societies	All MRONJ	Navigating the complexities and controversies of medication-related osteonecrosis of the jaw (MRONJ): a critical update and consensus statement	Mentioned but need more evidence	N/A	N/A	N/A
2024 (152)	Italia	Oral pathology and medicine	All MRONJ	Italian position paper (SIPMO-SICMF) on medication-related osteonecrosis of the jaw (MRONJ)	Mentioned but need more evidence	N/A	N/A	N/A
2022 (2)	America	OMFS	All MRONJ	American Association of Oral and Maxillofacial Surgeons’ Position Paper on Medication- Related Osteonecrosis of the Jaws-2022 Update	Mentioned but need more evidence	N/A	Mentioned but need more evidence	N/A
2020 (154)	Colombia	OMFS and osteoporosis	All MRONJ	Therapeutic approach and management algorithms in medication-related osteonecrosis of the jaw (MONJ): recommendations of a multidisciplinary group of experts	Suggested the use in the management of MRONJ in patients in the non-cancer setting	20 μg daily subcutaneous for 24 months	N/A	N/A

## 6 Discussion

While drug repurposing offers new possibilities, few repurposed drugs for managing MRONJ have reached clinical practise. Although this approach is faster and more cost-effective than traditional drug discovery, rushing into clinical trials can lead to risks of failure in later stages (156). A recent analysis found that fewer than 20% of hypotheses are successfully repositioned (157). Additional challenges include legal and regulatory obstacles, as well as issues related to pharmacology and dosing.

### 6.1 Patent and regulatory considerations

Intellectual property (IP) rights present challenges for drug repurposing. Securing IP protection for a newly repurposed medical use of an existing drug is possible if the new use is novel and inventive (158, 159). However, many repurposing opportunities are already documented in scientific literature, limiting patent protection options. Even when a drug shows promise, market entry can be delayed or blocked by IP conflicts (160). For off-patent drugs, limited return on investment makes industries reluctant to fund trials. Beyond patent issues, regional legal and regulatory obstacles hinder progress (161, 162). Regulatory incentives or formal guidance to encourage repurposing research are often lacking.

### 6.2 Drug safety and efficacy of TPTD

Pharmacological challenges arise when estimated effective drug concentrations cannot be clinically achieved through repurposing. Drugs designed for specific receptors or tissues may not have the same efficacy when used for other therapeutic purposes. Higher doses or drug interactions may be necessary to reach therapeutic levels, which could introduce off-target effects and unforeseen side effects. In patients with cancer, polypharmacy can further complicate treatment, leading to unexpected interactions. For instance, TPTD’s potential carcinogenic effects limit its use in patients with cancer, while PENTO may enhance blood flow, potentially increasing the risk of metastasis. The feasibility of repurposed treatments depends on the tolerability of side effects during administration.

None of the trials on TPTD or PENTO have determined whether drug suspension for established MRONJ is necessary during the interventions. In fact, there is a lack of consensus in the guidelines regarding the suspension of antiresorptive therapy for treating MRONJ (163). There is insufficient evidence to suggest that the benefits outweigh the risks to the primary disease; thus, recommendations should be interpreted cautiously (164). Drug suspension should be considered on a case-by-case basis in consultation with the prescribing clinician.

Additionally, dosage adjustments can escalate the risk of adverse events. Repurposing TPTD for MRONJ requires doses of 20 μg/d or 56.5 μg/week, the latter of which is not commonly used for osteoporosis treatment. Although trials have compared these dosing protocols (149), more evidence is needed to determine the safety and effectiveness. In addition, the staging of MRONJ varies among each patient, and there are significant differences in the extent and size of involvement. However, in the published results, the exploration of the optimal dosage is still ongoing, and there are no specific recommendations for patients with MRONJ at different stages yet.

### 6.3 Tailoring personalised protocol and delivery

With the ageing population, the demand for anti-resorptive and anti-angiogenic drugs is growing, along with the complexity of patient situations. The use of multiple medications and presence of accompanying diseases in vulnerable populations significantly challenge the management of MRONJ. Studies have shown that in the 3 d before chemotherapy, patients took an average of 9.6 concomitant medications, many of which affect drug metabolism and clearance (165). The interactions among different drugs make selecting appropriate medications even more difficult. Adding medications to prevent MRONJ may increase the risk of overuse, necessitating a more comprehensive and personalised treatment plan for this group of patients.

For patients unable to tolerate oral or injectable drugs, regional drug delivery may be a better option. Compared with systemic administration, applying drugs directly to the jawbone or at-risk areas could improve efficacy and reduce side effects. Further research into regional drug delivery methods should be prioritised.

Despite the severe clinical impact of MRONJ, effective prevention and treatment strategies remain limited. MRONJ poses significant challenges for patients undergoing anti-resorptive or anti-angiogenic therapies, particularly those with cancer. Current interventions, supported by low-level evidence, are not universally applicable and yield inconsistent outcomes across medical centres. With the rise of new cancer therapies and the complex health issues faced by patients with cancer, drug repurposing offers a potential solution for rapid and safe MRONJ management. By adopting drug-, disease-, and target-centric approaches, candidate drugs can be screened for personalised and precise treatment options.

### 6.4 Overall assessment and limitations

Based on existing evidence, PENTO appears to be the most promising option, particularly for cancer patients, owing to its favourable safety profile and lack of theoretical risk for promoting metastasis. Conversely, TPTD demonstrates significant potential for bone regeneration but is best reserved for non-oncologic osteoporotic patients due to contraindications in active malignancy. Despite these promising findings, a major evidence gap remains: there is a lack of large-scale RCTs directly comparing these protocols against the standard of care. Future research must prioritise establishing standardised dosing regimens and validating these findings in human cohorts to transition these therapies from experimental use to clinical guidelines.

Consequently, this review has several limitations. First, it relies primarily on data from small-sample clinical studies and animal models, as high-quality RCTs remain rare. Second, while the mechanistic hypotheses regarding TPTD and PENTO are theoretically well-detailed, they currently lack robust validation in human subjects. Third, due to inconsistent reporting in the primary sources, the discussion regarding specific adverse events and long-term safety profiles is limited. Finally, cost-effectiveness analyses and patient-reported outcomes were not addressed, as they were beyond the scope of this paper.

## 7 Conclusion

While the management of MRONJ is still evolving and pharmacological protocols have shown some effectiveness, their limitations pose risks, particularly for patients with cancer. Currently, TPTD and PENTO are used in the treatment of MRONJ, with PENTO potentially offering a safer and more effective option owing to its cAMP-mediated pathway and antioxidant effects. Given the complexity of systemic conditions and treatment protocols in cancer-related MRONJ, further research into repurposing additional therapeutic options, suitable for both prevention and treatment, is necessary.
